# Advancements in the investigation of chemical components and pharmacological properties of *Codonopsis*: A review

**DOI:** 10.1097/MD.0000000000038632

**Published:** 2024-06-28

**Authors:** Rui Chu, Yiquan Zhou, Chenjuan Ye, Rui Pan, Xiaomei Tan

**Affiliations:** a Chongqing College of Traditional Chinese Medicine, Chongqing, China; b Chongqing Academy of Chinese Materia Medica, Chongqing, China.

**Keywords:** biological activities, chemical components, *Codonopsis*, pharmacological effects

## Abstract

Species of the genus *Codonopsis* (Campanulaceae) have a long history of application, acclaimed for its edible and therapeutic attributes. Scholarly inquiries into *Codonopsis* span botany, phytochemistry, quality assurance, pharmacodynamics, and toxicity, revealing a rich and comprehensive body of knowledge. This study synthesizes information from esteemed scientific databases like SciFinder, PubMed, China National Knowledge Infrastructure, and Chinese herbal classics to create a thorough scientific conceptual and theoretical framework for *Codonopsis* research. In this article, the phytochemical composition includes saccharides, polyacetylenes, polyenes, flavonoids, alkaloids, lignans, terpenoids, and organic acids was summarized. To date, over 350 monomeric compounds have been isolated and identified from *Codonopsis*, with recent studies primarily focusing on polysaccharides, aromatic derivatives, lignans, and polyacetylenes. *Codonopsis* exhibits broad pharmacological activities across various systems, including immune, blood, cardiovascular, central nervous, and digestive systems, with no significant toxicity or adverse effects reported. The existing research, focusing on various extracts and active parts without identifying specific active molecules, complicates the understanding of the mechanisms of action. There is an urgent need to advance research on the chemical composition and pharmacological effects to fully elucidate its pharmacodynamic properties and the basis of its material composition. Such efforts are crucial for the rational development, utilization, and clinical application of this herb.

## 1. Introduction

The genus *Codonopsis*, belonging to the Campanulaceae family, mainly distributed throughout East and Southeast Asia. There are 39 species identified in China, among the more than 46 species.^[1]^ These species harbor chemical constituents of diverse structural types, predominantly including saccharides, polyacetylenes, polyenes, alkynes, flavonoids, alkaloids, lignans, phenylpropanoids, terpenoids, steroids, organic acids, and other. The dried root of 3 *Codonopsis* species: *Codonopsis pilosula* (Franch) Nannf., *Codonopsis pilosula* Nannf.var. modesta (Nannf.) L.T. Shen, and *Codonopsis tangshen* Oliv are utilized in traditional Chinese medicine and as dietary supplements, known as Codonopsis Radix (CR).^[2]^ It is cultivated extensively in the Shanxi, Gansu, Shaanxi, Hubei, and Sichuan Provinces, driven by considerable market demand. CR demonstrates substantial effects on various bodily systems, enhancing spleen and lung functions, nourishing blood, and promoting fluid production. Certain species within this genus are also significant food materials, widely used in China and Southeast Asia for tea, wine, soup, and other culinary purposes.

This study aims to consolidate the foundation for future research and application of *Codonopsis* by reviewing pharmacological and phytochemical research developments, drawing on global journal articles and databases like SciFinder, PubMed, China National Knowledge Infrastructure, and Chinese herbal classics.

The objective is to establish a robust scientific framework and theoretical underpinning for deepening the exploration of *Codonopsis*, evaluating its ethnopharmacological uses, identifying active components and their pharmacological actions, and improving its safety in clinical contexts, ultimately enhancing the scholarly substance and significance of our investigation.

## 2. Phytochemistry

To date, researchers have isolated and identified over 350 chemical compounds from *Codonopsis*,^[3]^ with key constituents being saccharides, polyacetylenes, polyenes, alkynes, flavonoids, alkaloids, lignans, phenylpropanoids, terpenoids, steroids, and organic acids.^[4]^

### 2.1. Saccharides

*Codonopsis* features a varied spectrum of saccharide components, encompassing monosaccharides, oligosaccharides, and polysaccharides. Within these, polysaccharides are deemed crucial active compounds, chiefly comprising pentoses, hexoses, and their derivatives. Arabinose is the foremost pentose, while glucose, galactose, and fructose are the leading hexoses. Additionally, mannitol is noteworthy as a significant derivative within these polysaccharide structures. Monosaccharides, including fructose, glucose, rhamnose, and galactose, coexist with oligosaccharides and polysaccharides, which are primarily structured from monosaccharide units and their derivatives.

Isolated and purified from *Codonopsis*, the fructan associated with β-(2→1) fructofuranosyl links, CPP-1, is a neutral polysaccharide.^[5]^ The water-soluble polysaccharide CPP from Codonopsis contains monosaccharides such as galactose, rhamnose, and arabinose.^[6]^ CPPS1 includes glucose, fructose, galactose, mannose, ribose, and arabinose.^[7]^ In CPPS3, the ratio of galactose to arabinose to rhamnose is 1.13:1.12:1.^[8]^ From Banqiao’s codonopsis *C tangshen* Oliv, 2 types of water-soluble polysaccharides, COP-I and COP-II, were isolated and purified. Mannitol, fructose, and glucose compose COP-I, a neutral oligosaccharide, while mannitol, fructose, glucose, and galactose are the constituents of COP-II, an acid heteropolysaccharide.^[9]^ Homogenized polysaccharide CPP 2–4, with a molecular weight of 3.9 × 10^4^ kDa was obtained from the bottom phase. The physicochemical properties and structural features confirmed it was a α-1,6-glucan.^[10]^ A homogeneous polysaccharide was extracted and purified from the root of *C pilosula* (Franch.) Nannf., revealing that it was composed of mannose, glucose, galactose, and arabinose.^[11]^

The monosaccharide composition of CLRP-1 and CLSP-1 includes Ara, Rha, Fuc, Xyl, Man, Gal, GlcA, and GalA, with molecular weights of 15.9 and 26.4 kDa, respectively. Structural analyses indicated that CLRP-1 and CLSP-1 are pectic polysaccharides, predominantly composed of 1,4-linked galacturonic acid, featuring extensive homogalacturonan regions and arabinogalactan types I and II as side chains.^[12]^ Pectic polysaccharides from the stems of *C pilosula* (CPSP-1) and *C tangshen* (CTSP-1) were isolated using ion exchange chromatography and gel filtration, with molecular weights of 13.1 and 23.0 kDa, respectively, characterized by extensive homogalacturonan and rhamnogalacturonan I regions.^[13]^

### 2.2. Polyacetylenes, polyenes, and their glycosides

The chemical components of polyenes and alkynes are widely distributed in the *Codonopsis* wall, such as lobetyol (1), lobetyolin (2),^[14]^ and lobetyolin (3).^[15]^ Three new linear C_14_ polyyne (=polyacetylene) glucosides, cordifolioidynes A–C (4–6) were isolated from the roots of *Codonopsis* cordifolioidea.^[16]^ Pilosulyne A–G (7–13) were isolated from the roots of *C pilosula*.^[17]^ Furthermore, the discovery of compounds is increasingly expanding, for instance, choushenpilosulyne A–C (14–16),^[18]^ codonopilodiynoside C–M (17–27), pratialin B (28), codonopiloenynenoside A–B (29–30),^[19,20]^ tetradeca-4*E*,8*E*,12*E*-triene-10-yne-1,6,7-triol (31), (+)-(6*R*,7*R*,12*E*)-tetradeca-12-en-10-yne-1,6,7-triol (32), (2*E*,6*E*)-octa-2,6-dien-4-ynoic acid(33), (*E*)-oct-6-en-4-ynoic acid (34), (+)-(2*R*,7*S*)-1,7-dihyrdroxy-2,7-cyclotetradeca-4,8,12-trien-10-yn-6-one (35),^[21]^ 9-(tetrahydropyran-2-yl)-non-trans-2,8-diene-4,6-diyn-1-ol (36),^[22]^ 9-(tetrahydropy ran-2-yl)-trans-non-8-ene-4,6-diyn-1-ol (37),^[23]^ pilosulinene A (38), pilosulinol A–B (39–40),^[24]^ tangshenyne A–B (41–42),^[25]^ (−)-(8*R*,9*R*,2*E*,6*E*,10*E*)-tetradeca-2,6,10-triene-4-yne-8,14-diol-9-β-d-glucopyranoside (43).^[26]^

**Figure 1.**
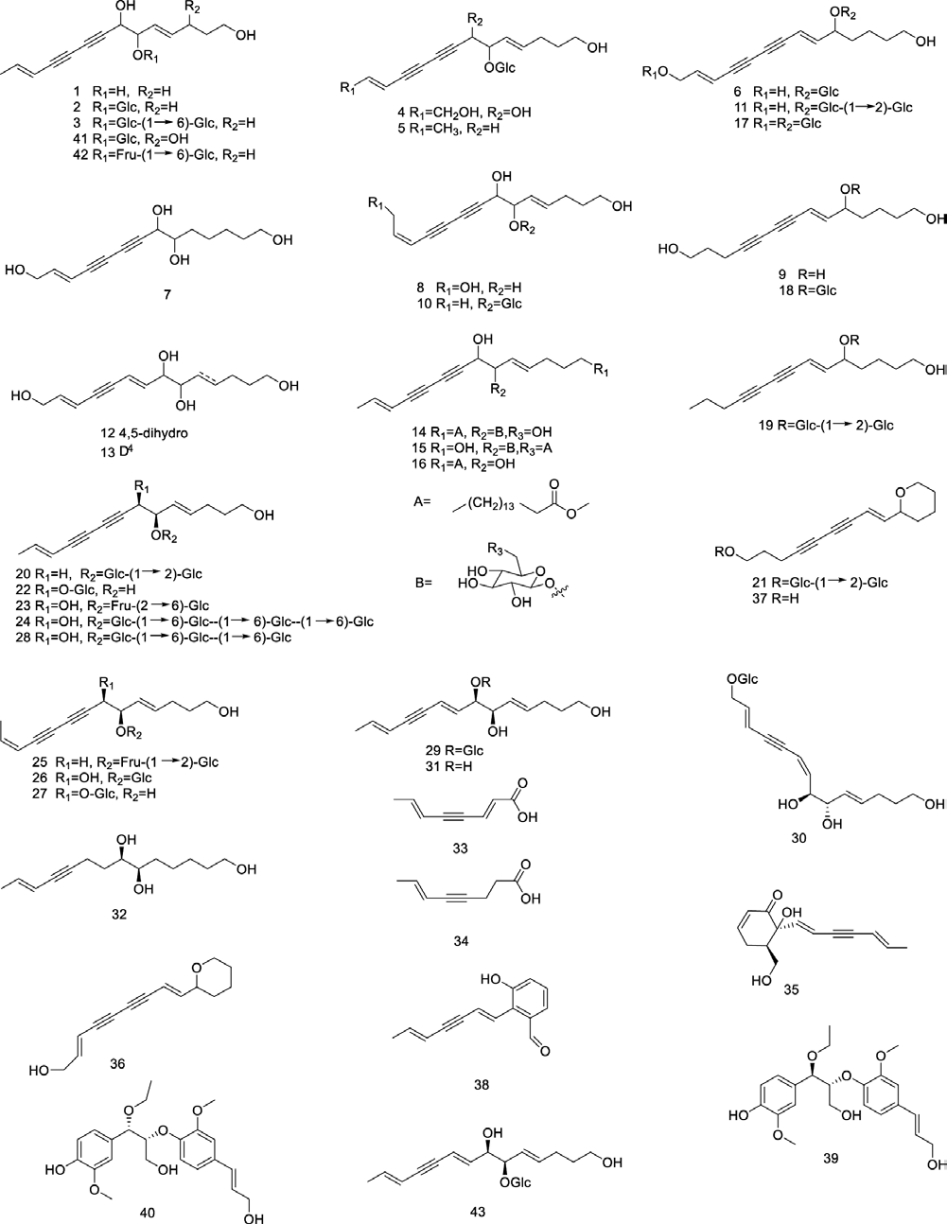
Structural of polyacetylenes, polyenes, and their glycosides from genus *Codonopsis*.

Additionally, it was discovered that Figure 1 provided a summary of the structures of the polyacetylenes, polyenes, and their glycosides extracted from *Codonopsis*.

### 2.3. Flavonoids and their glycosides

**Figure 2.**
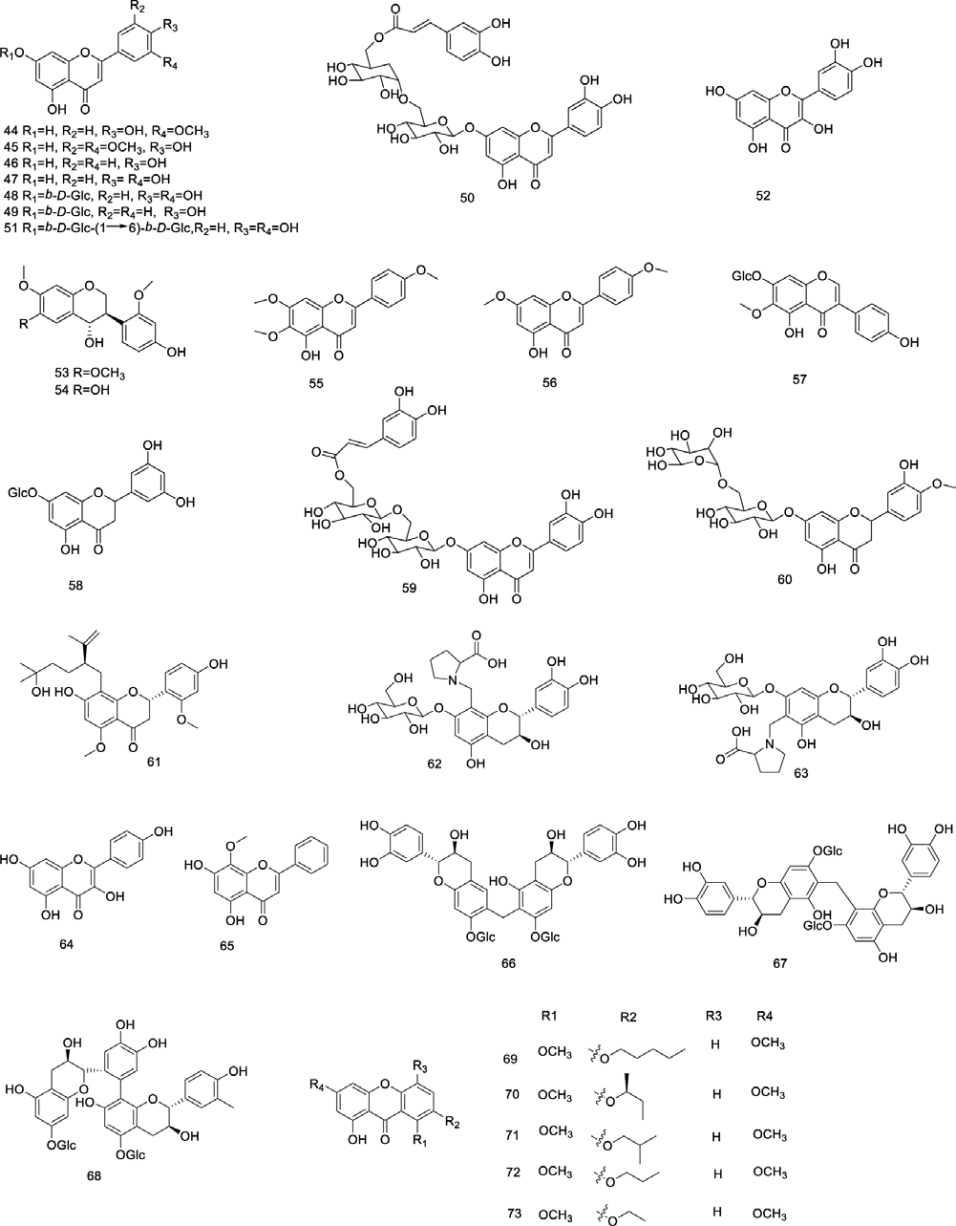
Structural of flavonoids and their glycosides from genus *Codonopsis*.

Flavonoids and their glycosides isolated from *Codonopsis*, are depicted in Figure 2, including chrysoeriol (44), tricin (45), apigenin (46), luteolin (47), luteolin-7-O-β-d-glucopyranoside (48), apigenin-7-O-β-d-glucopy ranoside (49), luteolin-7-O-β-d-glucopyranosyl-(1→6)-[(6‴-O-caffeoyl)]-β-d-glucopyranoside (50), luteolin-7-O-β-d-gentiobioside (51),^[27,28]^ quercetin (52), cordifoliflavane A–B (53–54), 5-hydroxy-4′,6,7-trimethoxy flavone (55), 5-hydroxy-4′,7-dimethoxy flavone (56), tectoridin (57), 5,7,3′,5′-tetrahydroxy-flavone-7-O-β-d-glucopyranoside (58),^[29]^ luteolin-7-O-[(6‴-caffeoyl)-β-d-glucopyranosyl-(1→6)]-β-d-glucopyranoside(59),^[30]^ hesperidin (60), neokurarinol (61),^[14]^ choushenflavonoid A–B (62–63),^[31]^ kaempferol (64),^[32]^ wogonin (65),^[33]^ choushenoside A–C (66–68).^[34]^ Furthermore, the isolation and structural elucidation of 5 new xanthones (69–73) have been described.^[35]^

### 2.4. Alkaloids

**Figure 3.**
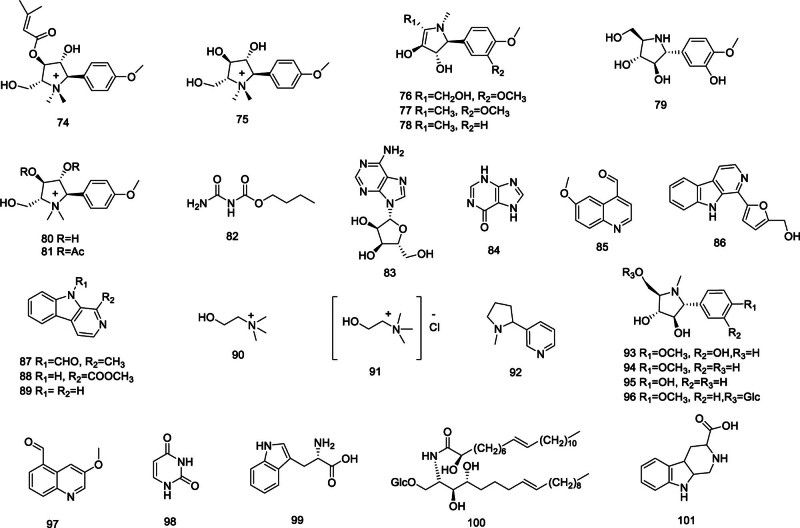
Structural of alkaloids from genus *Codonopsis*.

The alkaloids extracted from *Codonopsis* primarily include: codonopyrrolidium A–B (74–75),^[36,37]^ codonopsinol (76), codonopsine (77), codonopsinine (78), radicamine A (79),^[15,38]^ codotubulosine A–B (80–81),^[39]^ *n*-butyl allophanate (81), adenosine (83),^[26]^ hypoxanthine (84), 6-methoxy-quinoline-4-carbaldehyde (85),^[40]^ perlolyrine (86), *N*-9-formylHarman (87), 1-carbomethylcarboline (88), norharman (89),^[41]^ choline (90), choline chloride (91),^[29]^ nicotine (92), codonopsinol A–C (93–95), codonopiloside A (96), 6-methoxy-4-formyl quinolone (97),^[38]^ uracil (98), tryptophan (99),^[42]^ codonocerebroside A (100),^[43]^ 1,2,3,4-tetrahydro-β-carboline-3-carboxylic acid (101),^[44]^ and structure as shown in Figure 3.

### 2.5. Phenylpropanoids

**Figure 4.**
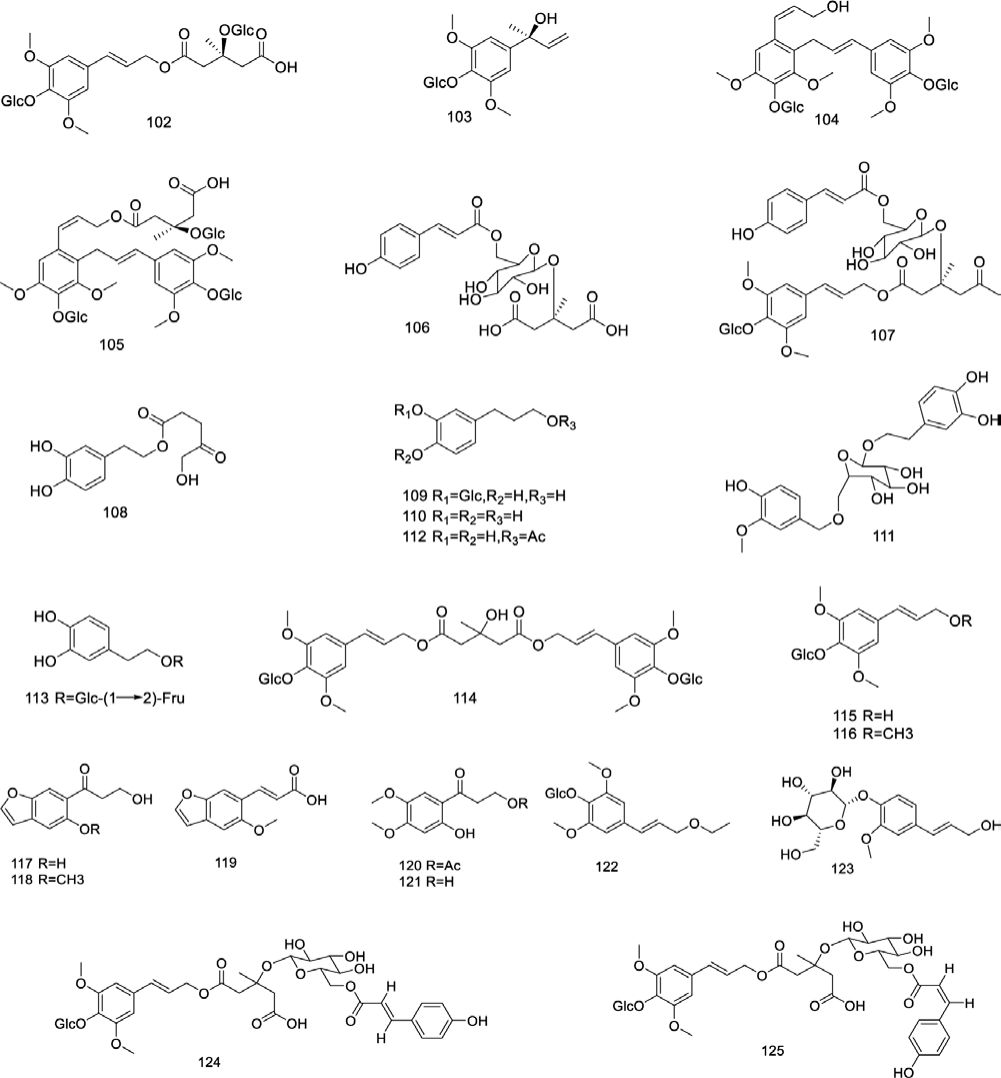
Structural of phenylpropanoids from genus *Codonopsis*.

Figure 4 illustrates the phenylpropanoids extracted from *Codonopsis* are primarily composed of tangshenoside I–VI (102–107),^[45]^ 3,4-dihydroxyphenethyl-5-hydroxy-4-oxopentanoate (108), 2-(3-O-β-d-glucopyranosyl-4-hydroxyphenyl) ethanol (109), 2-(3,4-dihydroxyphenyl)ethanol (110), 1′-O-β-d-(3,4-dihydroxypheny1)-ethyl-6′-O-vanilloyl-glucopyranoside (111), 4-(2-acetoxyethy1)-1,2-dihydroxy-benzene (112), and 3,4-dihydroxyphenylethano1-8-O-[β-d-apiofuranosyl(1→2)]-β-d-glucopyranoside (113).^[46]^ Additionally, newly identified compounds include tangshenoside VIII (114), syringin (115),^[47]^ methlysyringin (116),^[48]^ lanceolune A–C (117–119),^[49]^ cordifoliketone A–B (120–121),^[50]^ ethylsyringin (122),^[27]^ codonoside A (123), codonoside B (124),^[51]^ and coniferoside (125).^[26]^

### 2.6. Lignans

**Figure 5.**
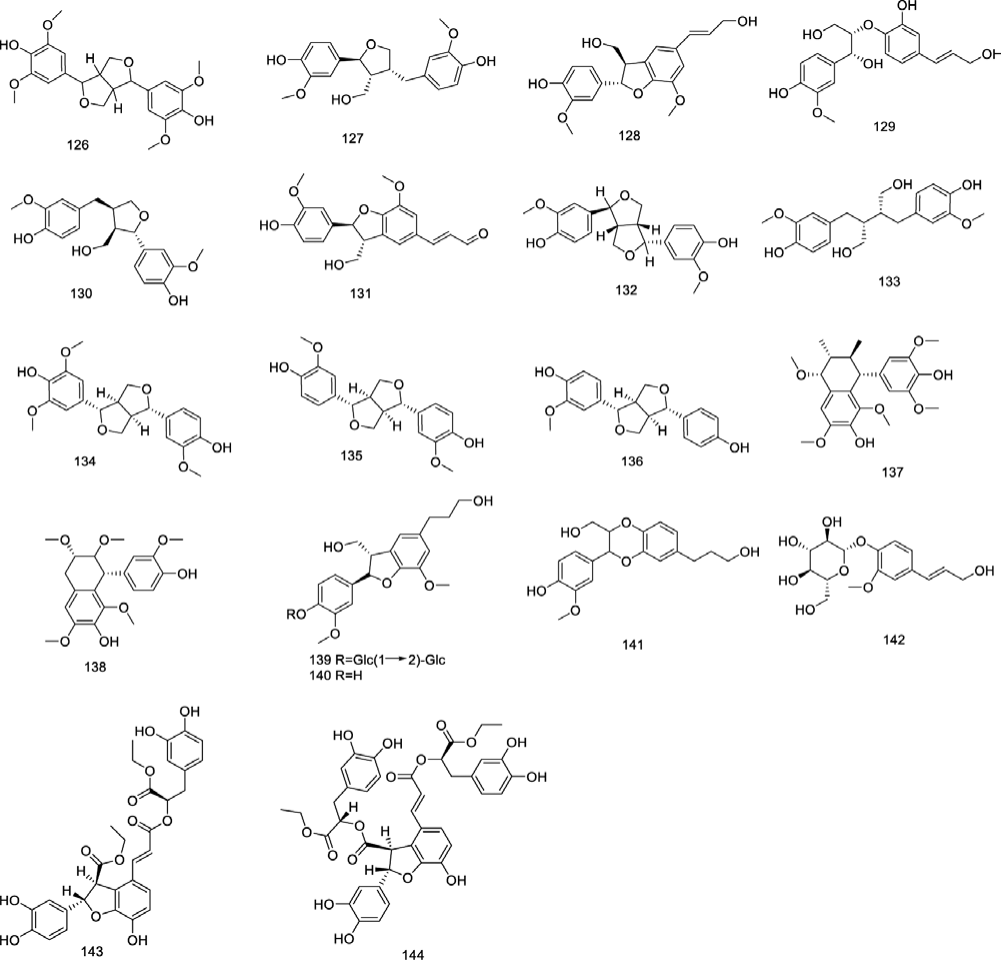
Structural of lignans from genus *Codonopsis*.

The lignan compounds in *Codonopsis* are characterized by syringaresinol (126),^[48]^ lariciresinol (127),^[52]^ dehydrodiconiferyl alcohol (128), (−)-(7*R*,8*S*,7′*E*)-3′,4-dihydroxy-3-methoxy-8,4′-oxyneoligna-7′-ene-7,9,9′-triol (129),^[23]^ lariciresinol (130), balanophonin (131), epipinoresinol (132), (−)-secoisolariciresinol (133), medioresinol (134), pinoresinol (135), (+)-demethoxypinoresinol (136), 4,4′-dihydroxy-3,3′,5,5′,7-pentamethoxy-2,7′-cyclolignane (137), (−)-ent-isolariciresinol (138), (−)-(7*R*,8*S*)-dihydrodehydrodiconiferylalcohol-4-O-β-d-glucopyranosyl-(1‴→2″)-β-d-glucopyranoside (139), (−)-(7*R*,8*S*)-dihydrodehydrodiconiferyl alcohol (140), (+)-(7*S*,8*S*)-3-methoxy-3′,7-expoxy-8,4′-neolignan-4,9,9′-triol (141),^[53]^ coniferoside (142),^[26]^ diethyl lithospermate (143), and diethyl litospermate B (144).^[54]^ These compounds are depicted in Figure 5.

### 2.7. Terpenoids and their glycosides

**Figure 6.**
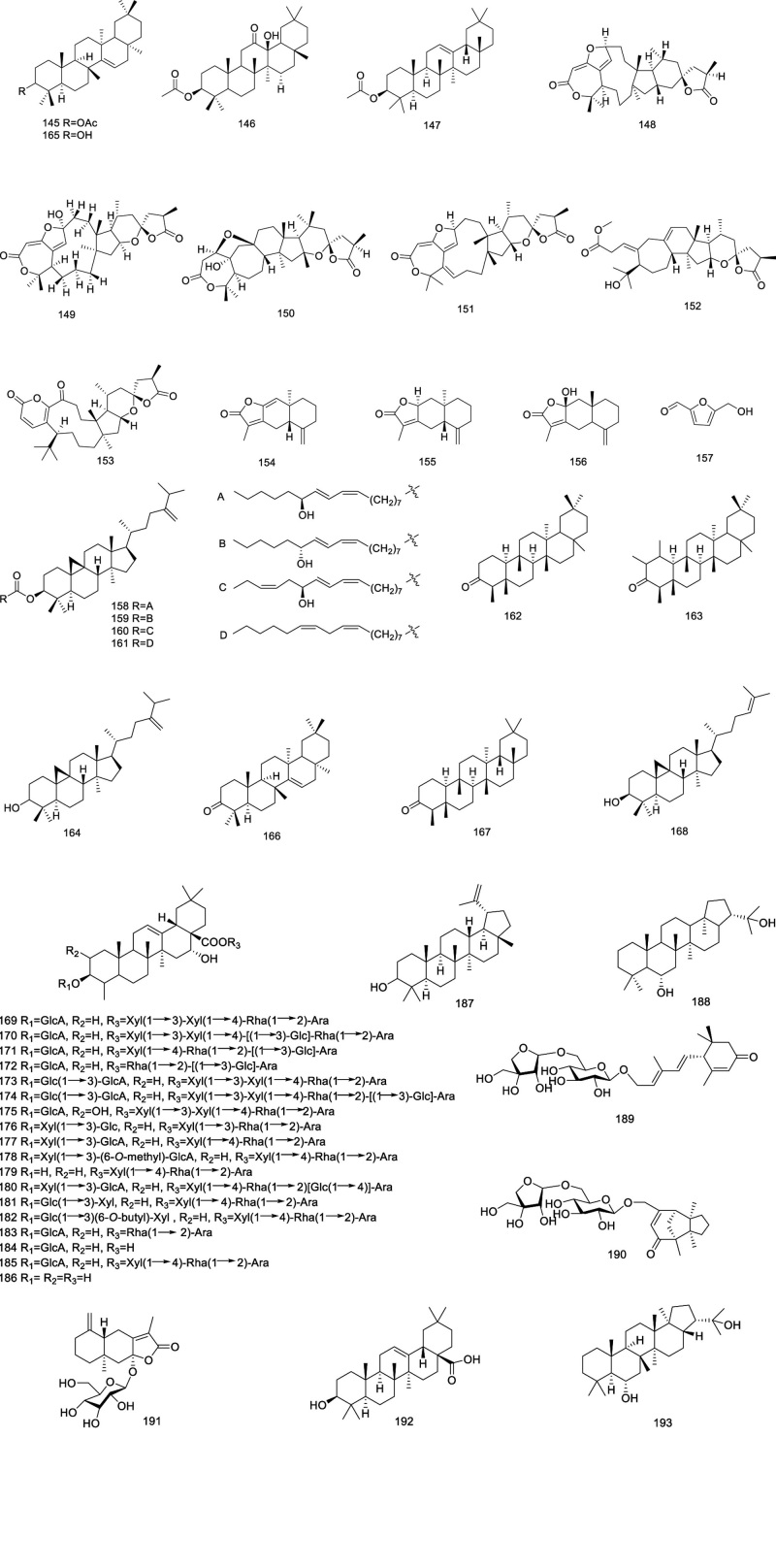
Structural of terpenoids and their glycosides from genus *Codonopsis*.

Terpenoids and their glycosides in *Codonopsis* as listed in Figure 6, comprising taraxeryl acetate (145), rubiprasin B (146),^[15]^ β-amyrin acetate (147), pseudolarolides E–P (148–151), pseudolarolides U–V (152–153),^[55]^ atraetylenolide I–III (154–156), 5-hydroxymethyl-2-furaldehyde (157),^[39]^ codonopilate A–C (158–160), 24-methylenecycloartanyllinolate (161), friedelan-3-one (162), 1-friedelan-3-one (163), 24-methylenecycloartan-3-ol (164),^[35,56]^ taraxerol (165), taraxerone (166), friedelin (167),^[57]^ cycloartenol(168),^[58]^ lancemasides A–G (169–175),^[59,60]^ codonoposide (176), codonolaside (177), codonolaside I–V (178–182),^[61]^ foetidissimoside A (183), acid-3-β-d-glucopyranosyl methyl furfural (184), echinocystic acid-3-O-β-d-glucuronopyranoside (185),^[15]^ echinoeystie acid (186), lupeol (187), hopane-6α-22-diol (188), codonopsesquiloside A–C (189–191),^[62]^ oleanolic acid (192),^[63]^ and zeorin (193).^[64]^

### 2.8. Steroids and their glycosides

**Figure 7.**
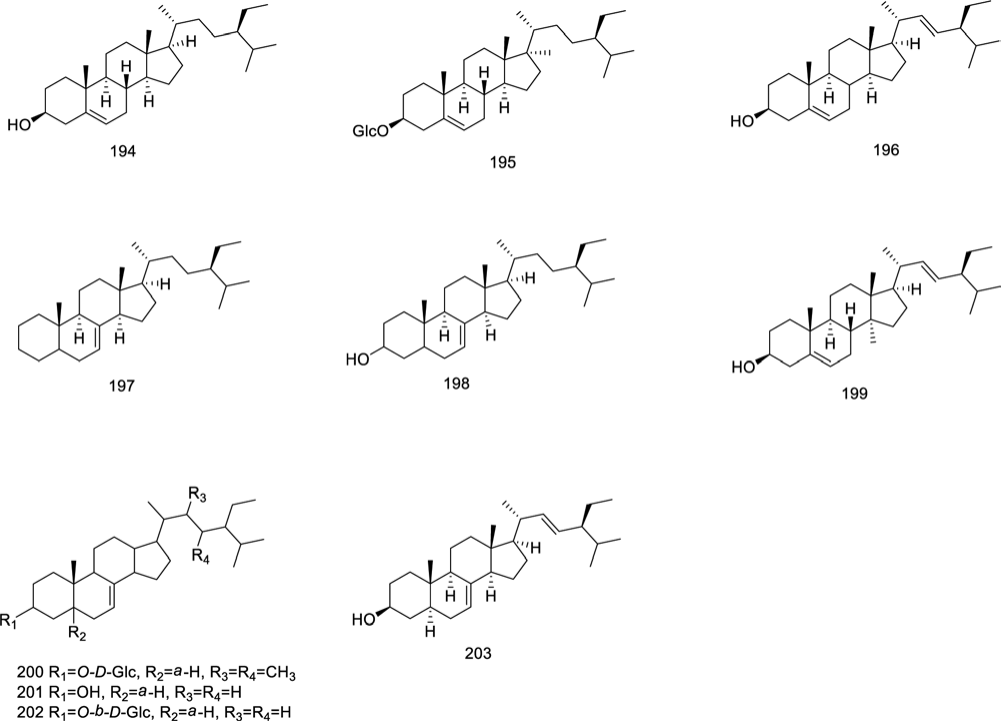
Structural of steroids and their glycosides from genus *Codonopsis*.

As shown in Figure 7, the steroid compounds isolated from *Codonopsis* are relatively few, including *β*-sitosterol (194),^[65]^ *β*-daucosterol (195), stigmasterol (196), stigmast-7-ene-3-one (197), stigmast-7-en-3-ol (198),^[35,66]^ Δ5, 25-stigmasterol (199), *α*-spinatsrol-*β*-d-glucoside (200), Δ7-stigmasterol (201), Δ7-stigmasteryl glucoside (202),^[67]^ and *α*-spinasterol (203).^[32]^

### 2.9. Phenolic acids and organic acids

**Figure 8.**
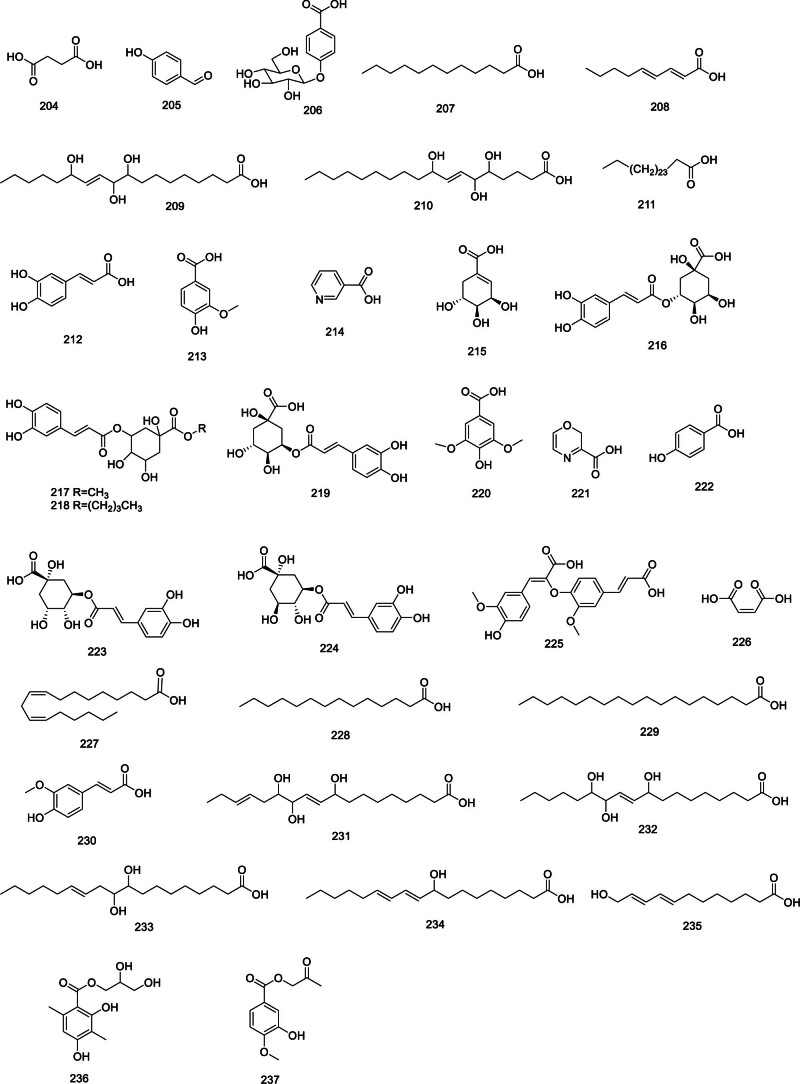
Structural of phenolic acids and organic acids from genus *Codonopsis*.

The variety of phenolic and organic acids in *Codonopsis* is extensive, encompassing succinic acid (204), 4-hydroxybenzaldehyde (205), 4-(*β*-D-glucopyranosyl)-benzoic acid (206), lauric acid (207), 2,4-nonadlenic acid (208), 9,10,13-trihydroxy-(E)-11-octadecenoic acid (209), 5,6,9-trihydroxyoctadec-7-enoicacid (210), heptacosanoic acid (211),^[29]^ caffeic acid (212), vanillic acid (213), nicotinic acid (214),^[67]^ shikimic acid (215), 3-O-caffeoylquinic acid (216), 3-O-caffeoylquinic acid methyl ester (217), 3-O-caffeoylquinic acid butyl ester (218),^[32]^ 5-O-caffeoylquinic acid (219), syringic acid (220), codopiloic acid (221), 4-hydroxybenzoic acid (222), chlorogenic acid (223),^[27]^ neochlorogenic acid (224), 8-O-4′diferulic acid (225), maleic acid (226), linoleic acid (227), myristic acid (228), stearic acid (229),^[68]^ ferulic acid (230),^[69]^ 9,12,13-trihydroxy-10,15-octadecadienoic acid (231), 9,12,13-trihydroxy-10-octadecenoic acid (232), 9,10-dyhydroxy-12-octadecenoic acid (233), 9-hydroxy-10,12-octadecadienoic acid (234), (8*E*,10*E*)-12-hydroxydodeca-8,10-dienoic acid (235),^[70]^ and introduces 2 novel aromatic derivatives: 2,3-dihydroxypropyl-2,4-dihydroxy-3,6-dimethylbenzoate (236) and 2-oxopropyl 3-hydroxy-4-methoxybenzoate (237),^[71]^ summarized in Figure 8.

### 2.10. Others

**Figure 9.**
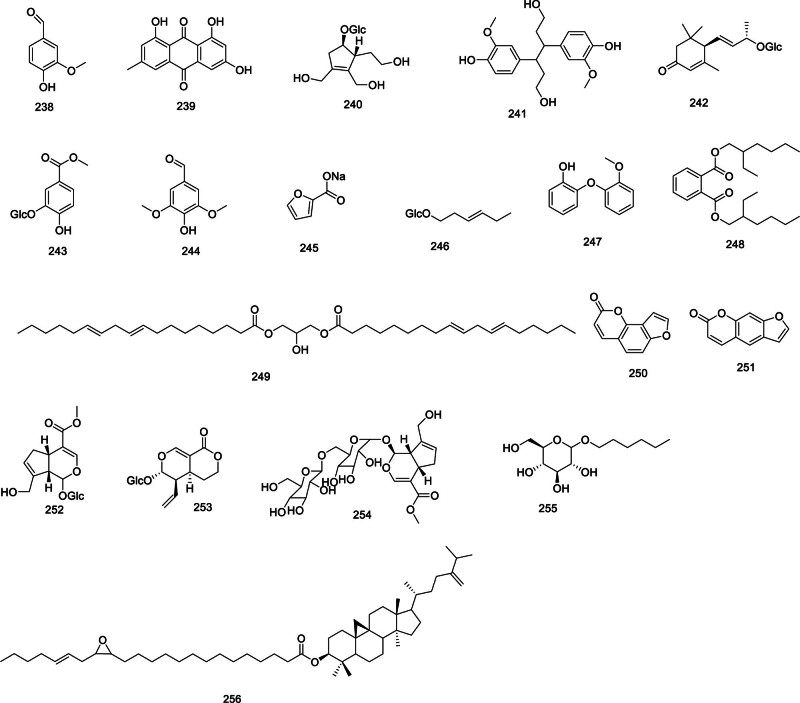
Structural of other compounds from genus *Codonopsis*.

Furthermore, *Codonopsis* features a variety of volatile oils,^[72–74]^ encompassing alcohols, olefins, terpenes, fatty acids and fatty acid esters. The plant has yielded compounds such as vanillin (238), emodin (239), eucommioside II (240), 1,6-hexanediol-3,4-bis(4-hydroxy-3-methoxyphenyl) (241), (6R,9S)-3-oxo-α-ionol-*β*-D-glucopyranoside (242),^[15]^ woodorien (243), syringaldehyde (244), 2-furan sodium salt (245), (Z)-3-hexenyl-*β*-D-glucopyranoside (246), O-(O-methoxyphenoxy) phenol (247), bis-(2-ethylhexyl)-phthalate (248),^[57]^ 1,3-linolein-2-olein (249),^[29]^ angelicin (250), psoralen (251), geniposide (252),^[75]^ sweroside (253),^[65]^ genipingentiobioside (254),^[76]^ hexyl-*β*-d-glucopyranoside (255)^[66]^ from *Codonopsis* has been reported. A new cycloartanyl ester named codonopilate D (256), was isolated from the roots of *C pilosula* (Franch.) Nannf,^[77]^ illustrated in Figure 9.

## 3. Pharmacological effects

Recent pharmacological studies have revealed that *Codonopsis* provides numerous health benefits, including regulating blood glucose levels, enhancing immunity, promoting hematopoietic activity, offering anti-hypoxic and anti-stress properties, reducing fatigue, providing antiaging benefits, managing gastric motility, protecting gastrointestinal mucosa, and preventing ulcer formation.

### 3.1. Regulation of body immunity

*Codonopsis* polysaccharides have been shown to enhance the phagocytic activity of alveolar macrophages, which is often impaired in chronic obstructive pulmonary disease, and to reduce inflammatory responses.^[78]^ As a potent immunomodulator, it influences T cell distribution, maintaining T cell balance in murine models, and reducing the incidence of sepsis after cecal ligation and puncture.^[79–81]^ Additionally, the selenium component in *Codonopsis* polysaccharide augments the phagocytic index of peritoneal macrophages and stimulates the secretion of TNF-β and IL-6, while its extracellular polysaccharide promotes macrophage activation, thereby hindering cancer cell proliferation and migration.^[82]^ The immunomodulatory properties of 2 *Codonopsis* pectin polysaccharides, RCAP-1 and RCAP-2,^[83]^ are significant. In an accelerated aging animal model, the immunomodulatory influence of Codonopsis pectin polysaccharide was investigated, revealing its potential to activate T cells through the TCR/CD28 signaling pathway.^[84]^ Additionally, a methanol extract of *Codonopsis* ginseng exhibits anti-inflammatory attributes and modulates the macrophage-mediated immune response.^[85]^ Additionally, sulfuric acid esterification has been reported to enhance the immunological, antioxidative, and hepatoprotective effects of *C pilosula* polysaccharides (CPPS).^[86,87]^

The FRAP and ABTS assays demonstrated that CPP 2–4 exhibit potent antioxidant activity in a dose-dependent manner. Additionally, CPP 2–4 curtailed NO release in RAW264.7 cells stimulated by lipopolysaccharide, demonstrating a pronounced anti-inflammatory effect,^[10]^ which is corroborated by additional research.^[88]^ Total saponins from *Codonopsis*, key bioactive constituents of the plant, exhibit anti-inflammatory, antioxidant, anti-ulcer, and immunomodulatory effects, and provide protection against ulcerative enteritis.^[89]^ CPPS afford lung protection in LPS and *Escherichia coli*-induced ALI mouse models, indicating their potential as therapeutic agents for ALI.^[90]^ Inulin, a principal bioactive component from *C pilosula*, functions as a medicinal prebiotic, bolstering mucosal immunity, exerting anti-inflammatory effects, and modulating the microbiota.^[91]^ A homogenous polysaccharide from *C pilosula* diminishes the expression of inflammatory markers such as TLR4, NF-κB, TNF-α, and IL-6 in cells, indicating its anti-inflammatory properties may stem from the inhibition of the TLR4/NF-κB pathway.^[92]^ Moreover, *C pilosula* oligosaccharides, as significant immunomodulatory compounds in *C pilosula*, show promise for development as immunomodulators in medicinal or functional food sectors.^[93]^

### 3.2. Neuroprotection

Neuroprotection involves preserving nerve cells, enhancing memory and learning, and reducing Alzheimer’s disease (AD) symptoms. Lancemaside and a water-extracted *Codonopsis* are shown to repair injured neurons, diminish Tau protein phosphorylation, elevate protein phosphatase 2 activity, and suppress AChE activity.^[60]^ In PC12 cells, codonopsis alkaloids enhance mitogen-activated protein kinase phosphorylation and neurite outgrowth.^[94]^ Additionally, total saponins from Codonopsis improve the survival of cerebral astrocytes after ischemia-reperfusion injury in rats.^[95]^ Research also indicates that *Codonopsis* polysaccharides might boost learning and memory in lead-poisoned mice by enhancing lipid peroxidation and neutralizing free radicals in the brain.^[96]^ Furthermore, *Codonopsis* polysaccharides could improve brain function in model rats by influencing the Nrf2 pathway.^[97]^ Other research suggests that *Codonopsis* polysaccharide principally activates PP2A to prevent the hyperphosphorylation of AD-characteristic tau protein.^[98]^ The 70% ethanol-water extract of *C pilosula* demonstrates neuroprotective effects in vitro through antioxidant activities, inhibition of BAX and Caspase-3 expression, thus protecting mouse hippocampal neuron HT22 cells.^[99]^

EPP and POL from CR positively impact learning and memory in mice with scopolamine-induced memory deficits, indicating CR’s potential as a promising agent for the prevention and enhancement of learning memory.^[100]^

### 3.3. Controlling blood lipids and blood sugar

The water extract of *Codonopsis* is shown to decrease blood glucose levels, inhibit aldose reductase activity, and mitigate diabetes progression. *Codonopsis* polysaccharide effectively reduces blood glucose in diabetic mice by inhibiting gluconeogenesis, boosting glycogen synthesis, and improving insulin sensitivity.^[101]^ *Codonopsis* CLPS also counters insulin resistance triggered by a high-fat/high-sugar diet, leveraging its antioxidative properties,^[102]^ while its neutral polysaccharide markedly boosts insulin secretion in INS-1 cells and decreases blood glucose in type 2 diabetic mice.^[103]^

Oral intake of PCL effectively counterbalances the negative impacts of a high-fat diet, including weight gain, hepatic steatosis, mesenteric adipocyte hypertrophy, and impaired glucose/lipid metabolism, and it also supports mammary gland development and lactogenesis.^[104]^ CPPS encourage osteogenic differentiation and inhibit lipogenic differentiation in rat bone marrow stem cells via β-catenin activation.^[105]^ The hypoglycemic effects and gut microbiome modulation of *C pilosula* crude polysaccharides were examined in a type 2 diabetes mellitus mouse model induced by a high-fat diet and streptozotocin.^[106]^ Alkaloid extract from CR notably reduces hepatic lipid accumulation in a nonalcoholic fatty liver disease mouse model prompted by an a high-fat diet, curtailing abnormal weight gain, liver damage, and levels of serum triglycerides, total cholesterol, and low-density lipoprotein, while elevating high-density lipoprotein levels.^[107]^

### 3.4. Protecting gastrointestinal mucosa and anti-ulcer effects

*Codonopsis* extract is pharmacologically effects in treating gastric ulcers, enhancing intestinal motility, and improving digestive functions. Its water extract acts as a laxative in constipated mice.^[108]^ Superfine powder of *Codonopsis* safeguards the gastric mucosa in rats with gastric ulcers.^[109]^ Flavonoids from *Codonopsis* facilitate cell migration in small intestinal epithelial cells, countering the migration inhibition caused by DFMO or 4-AP and potentially increasing cellular arginine through polyamine signaling pathway modulation.^[110]^ *Codonopsis* increases prostaglandin levels, reduces gastrin-induced acid secretion, stimulates the synthesis and release of epidermal growth factor from the gastric mucosa, and protects against alcohol-induced gastric mucosal damage.^[111]^ *Codonopsis* polysaccharide augments the thickness of the gastric mucosa and stomach wall, encourages intestinal villi growth, enhances intestinal peristalsis and digestive function, and modulates colonic microbiota in colitis mice.^[112]^

Total saponins of *Codonopsis* significantly protects against ulcerative damage in the colonic mucosa of UC rats, exhibiting a dose-dependent effect; this is likely due to anti-lipid peroxidation and inhibition of the NF-κB signaling pathway, which regulates inflammatory factor release.^[113]^ *Codonopsis* inulin-type fructan CP-A ameliorates TNBS-induced ulcerative colitis.^[114]^ Nulin-type fructan from *C pilosula* roots defined as a polydisperse carbohydrate consisting mainly of β-(2-1) fructosyl-fructose links has potential in stimulating the growth of *Prevotella* and *Faecalibacterium* as well as *Bifidobacterium* of human gut microbiota.^[115]^

### 3.5. Enhancing hematopoietic function and cardiovascular protection

*Codonopsis* extracts are employed to treat heart failure, augment hematological function, regulate blood cell development and growth, and inhibit platelet aggregation.^[116]^ They can also reduce cardiomyocyte apoptosis and mitigate damage to the insulin-like growth factor II receptor pathway.^[117]^ An aqueous solution of *Codonopsis* offers protection against myocardial ischemia/reperfusion injury by decreasing left ventricular end-diastolic pressure, curbing the elevation of MDA, LDH, and CK, and enhancing the activities of Superoxide Dismutase (SOD), GSH-Px, Na^+^-K^+^-ATP, and Ca^2+^-ATP.^[118]^ Moreover, *Codonopsis* extract can improve chronic heart failure symptoms in rats, increase the maximum rate of rise/fall in left ventricular pressure, and delay the onset and progression of chronic heart failure.^[119]^ In heart failure mice, Codonshen granules reduce the duration of calcium transient decline, elevate ejection fraction, decrease left ventricular brachyaxis size, and increase calcium transient peak.^[120]^ Methanol extract from *Codonopsis* has been shown to maintain normal blood oxygen levels, reduce platelet activation-induced clotting, and modulate Lyn kinase activation.^[121]^ Ethanol extract of *Codonopsis* rotunda significantly lowers whole blood and plasma viscosity, red blood cell aggregation index, and hematocrit in rat models with Qi-deficiency type blood stasis syndrome.^[122]^ Additionally, a water extract of *Codonopsis* Rotunda can inhibit red blood cell hemolysis,^[123]^ and used to assist patients recovering from hematopoietic stem cell transplantation.^[124]^ Moreover, *Codonopsis* polysaccharide may delay X-ray-induced aging of hematopoietic stem cells through the p53–p21 signaling pathway.^[125]^ Other studies have found that *Codonopsis* lanceolata and its active compound Tangshenoside I mitigate skeletal muscle atrophy by modulating the PI3K/Akt and SIRT1/PGC-1α pathways.^[126]^

### 3.6. Antitumor effects

The water extract of *Codonopsis* can induce apoptosis in HL-60 cells,^[62]^ and *Codonopsis* saponins effectively inhibit the proliferation of human hepatoma HepG2 and SMMC-7721 cells.^[127]^ Water-soluble polysaccharides, CPP1a and CPP1c, enhance apoptosis in HepG2 cells by increasing the Bax/Bcl-2 ratio and activating caspase-3, also showing cytotoxic effects against cervical cancer Hela cells and gastric cancer MKN45 cells.^[128]^ Codonopsis pectin polysaccharide significantly impacts human lung adenocarcinoma A549 cells, showing notable cytotoxic effects.^[129]^ Moreover, *Codonopsis* acid polysaccharide is identified as a promising agent in inhibiting tumor metastasis by reducing the invasion, migration, and adhesion of human ovarian epithelial tumor HO-8910 cells.^[130]^ CR demonstrates anti-gastric precancerous lesion effects by mitigating gastritis injury and selectively inhibiting the proliferation of gastric cancer cells over normal cells.^[131]^ Lobetyolin targets cell proliferation and prompts apoptosis by downregulating ASCT2 in gastric cancer cytotechnology.^[132]^ Luteolin, an active component of *C pilosula*, exerts an anti-hepatocellular carcinoma effect via AKT- or MAPK-JNK signaling-mediated ESR1.^[133]^ *C pilosula* glucofructan, used as an immunopotentiator in previous research, has been shown to effectively inhibit tumor growth in mice.^[134]^ *C pilosula* may suppress hepatocellular carcinoma growth by targeting CDK1 and influencing the PDK1/β-catenin signaling pathway, which restricts cell epithelial–mesenchymal transition and diminishes cell stemness, highlighting its potential therapeutic value in liver cancer treatment.^[135]^

### 3.7. Antiaging and antioxidant effects

The water extract of *Codonopsis* counteracts d-galactose-induced aging in mice by reducing serum ALT and ALP levels, likely through miRNA target modulation.^[136]^ Total saponins from the Codonopsis stems and leaves exhibit concentration-dependent antioxidant activity.^[137]^ Total flavonoids from wild Xinjiang Codonopsis increase SOD activity in mouse serum and liver, reduce MDA content, extend weight-bearing swimming time, and demonstrate notable antioxidant and anti-fatigue effects.^[138]^ Selenated Codonopsis polysaccharide protects RAW264.7 cells from H_2_O_2_-induced oxidative damage and may enhance male reproductive function via the Keap1-Nrf2/ARE signaling pathway.^[139]^ Codonginseng polysaccharide decreases serum ALT, AST, TNF-α, and MDA levels while boosting SOD and GSH-Px activities, showing strong antioxidant and hepatoprotective properties in vivo and in vitro.^[87]^ *C pilosula* emerges as a promising agent for treating ulcerative colitis at optimal concentrations. Its therapeutic effects may involve alleviating colonic inflammation, correcting metabolic disorders, enhancing antioxidant capacity, and notably inhibiting the PI3K/Akt pathway.^[140]^

### 3.8. Antibacterial and antiviral properties

Recent studies highlight the antibacterial and antiviral capabilities of *Codonopsis*. In vitro research shows that the ethanol extract of *Codonopsis* significantly inhibits common pathogens, including *Staphylococcus aureus, Bacillus subtilis, Bacillus anthracis, Escherichia coli*, and *Salmonella typhi*.^[141]^ Additionally, *Codonopsis* polysaccharide reduces IFN-β expression and curtails the virulence of the duck hepatitis A virus,^[142]^ and it shows potential in mitigating liver fibrosis by modulating TLR4/NF-κB and TGF-β1/Smad3 signaling pathways, suggesting its therapeutic application in treating liver fibrosis.^[143]^

## 4. Summary and prospect

The genus *Codonopsi*s, renowned for its long-standing medicinal and culinary uses in China, has been extensively studied for its chemical components and wide-ranging pharmacological effects. This review offers a comprehensive overview of Codonopsis’s phytochemical profile and pharmacological mechanisms, drawing on respected scientific databases to evaluate its ethnopharmacological applications and future research directions.

Despite the thorough investigation into Codonopsis’s phytochemistry, with over 350 chemical constituents identified, the focus on various extracts and active components, which include a multitude of compounds with potential synergistic or antagonistic interactions complicates the understanding of its pharmacological actions. While this approach provides insight into the herb’s overall therapeutic potential, it obscures the specific functions and interactions of individual molecules.

This limitation impedes a full exploration of Codonopsis’s pharmacological mechanisms and presents challenges in establishing quality markers. There is an urgent need to deepen research into its chemical composition and pharmacological effects to elucidate its pharmacodynamic properties and the foundation of its material composition comprehensively. Future studies should employ a targeted strategy to identify and characterize pivotal active molecules, incorporating bioactivity-guided fractionation, structure-activity relationship studies, molecular docking, computational modeling, and in vivo pharmacokinetics and pharmacodynamics investigations. Focusing on pinpointing and detailing specific active compounds within *Codonopsis* is crucial for its rational development, utilization, and clinical application. Moreover, this research offers a valuable basis for uncovering new drug leads from *Codonopsis*, potentially enhancing public health and propelling the advancement of natural product-based drug discovery.

## Author contributions

**Data curation:** Rui Chu.

**Formal analysis:** Rui Chu.

**Funding acquisition:** Yiquan Zhou, Xiaomei Tan.

**Investigation:** Rui Pan.

**Project administration:** Yiquan Zhou.

**Resources:** Yiquan Zhou.

**Validation:** Chenjuan Ye.

**Writing – original draft:** Rui Chu.

**Writing – review & editing:** Xiaomei Tan, Rui Chu.
